# Shoots and Turions of Aquatic Plants as a Source of Fatty Acids

**DOI:** 10.3390/molecules29092062

**Published:** 2024-04-29

**Authors:** Maciej Strzemski, Lubomir Adamec, Sławomir Dresler, Barbara Mazurek, Katarzyna Dubaj, Piotr Stolarczyk, Marcin Feldo, Bartosz J. Płachno

**Affiliations:** 1 Department of Analytical Chemistry, Medical University of Lublin, 4a Chodzki St., 20-093 Lublin, Poland; slawomir.dresler@umlub.pl; 2 Department of Experimental and Functional Morphology, Institute of Botany of the Czech Academy of Sciences, Dukelská 135, CZ-379 01 Třeboň, Czech Republic; lubomir.adamec@ibot.cas.cz; 3 Department of Plant Physiology and Biophysics, Institute of Biological Sciences, Faculty of Biology and Biotechnology, Maria Curie-Skłodowska University, 19 Akademicka St., 20-033 Lublin, Poland; 4 Analytical Department, New Chemical Syntheses Institute, 13A Tysiąclecia Państwa Polskiego Ave., 24-110 Puławy, Poland; barbara.mazurek@ins.lukasiewicz.gov.pl; 5 Department of Basic Medical Sciences, Faculty of Medical and Health Sciences, Casimir Pulaski Radom University, 27 Boleslawa Chrobrego Str., 26-600 Radom, Poland; k.dubaj@urad.edu.pl; 6 Department of Botany, Physiology and Plant Protection, Faculty of Biotechnology and Horticulture, University of Agriculture in Kraków, 29 Listopada 54 Ave., 31-425 Cracow, Poland; piotr.stolarczyk@urk.edu.pl; 7 Department of Vascular Surgery, Medical University of Lublin, Staszica 11 St., 20-081 Lublin, Poland; martinf@interia.pl; 8 Department of Plant Cytology and Embryology, Institute of Botany, Faculty of Biology, Jagiellonian University in Kraków, 9 Gronostajowa St., 30-387 Krakow, Poland

**Keywords:** aquatic plants, *Aldrovanda*, *Utricularia*, *Myriophyllum*, *Stratiotes*, turions, essential fatty acid, GC-MS analysis

## Abstract

Background: Fatty acids are essential for human health. Currently, there is a search for alternative sources of fatty acids that could supplement such sources as staple crops or fishes. Turions of aquatic plants accumulate a variety of substances such as starch, free sugars, amino acids, reserve proteins and lipids. Our aim is to see if turions can be a valuable source of fatty acids. Methods: Overwintering shoots and turions of aquatic carnivorous plants were collected. The plant material was extracted with hexane. The oils were analyzed using a gas chromatograph with mass spectrometer. Results: The dominant compound in all samples was linolenic acid. The oil content was different in turions and shoots. The oil content of the shoots was higher than that of the turions, but the proportion of fatty acids in the oils from the shoots was low in contrast to the oils from the turions. The turions of *Utricularia* species were shown to be composed of about 50% fatty acids. Conclusions: The turions of *Utricularia* species can be used to obtain oil with unsaturated fatty acids. In addition, the high fatty acid content of turions may explain their ability to survive at low temperatures.

## 1. Introduction

### 1.1. The Roles and Sources of Unsaturated Fatty Acids

Unsaturated FAs, such as oleic (18:1), linoleic (18:2), and α-linolenic (18:3) acids, play an important role in plants and are also economically relevant traits of oil crops [1]. As precursors of vitamins, cofactors, or other biologically active molecules, fatty acids are essential for human health. However, the correct ratio of these acids in the human diet must be maintained for proper health. Disturbance in these proportions may result in various diseases [2]. Thus, it is extremely important to source food from which these acids are taken up in the right proportions. The sources of these FAs are mainly staple crops, especially the following oilseed crops: peanut (*Arachis hypogaea* L.), sunflower (*Helianthus annuus* L.), flax (*Linum usitatissimum* L.), rapeseed (*Brassica napus* L.), camelina (*Camelina sativa* L.), cotton (*Gossypium hirsutum* L.), safflower (*Carthamus tinctorius* L.), and castor bean (*Ricinus communis* L.) [3,4]. Currently, there is a search for alternative sources of FAs that could supplement or even replace such usual sources as common crops or fishes [5,6,7,8,9].

### 1.2. Turions of Aquatic Plants

Turions (winter buds) are vegetative dormant storage organs formed by perennial aquatic plants in subtropical to subarctic zones in response to unfavorable ecological conditions (low temperature, short days, low irradiance) at the beginning of autumn. They are produced in at least 14 genera of aquatic vascular plants, especially in submerged and free-floating species [10]. Turions are formed by the condensation of apical leaf nodes and shortened internodes with modified shortened leaves or scales. The turions of all species usually overwinter at the bottom of aquatic habitats in darkness and hypoxic or anoxic conditions [10,11]. 

Turions exhibit low rates of metabolism. Their aerobic respiration rate is several times lower per unit fresh weight (FW) or dry weight (DW) than that of summer leaves or shoots of the same or similar aquatic species [10,12,13]. Moreover, the dry matter content is usually between 18 and 39% in mature turions and is thus 2.5–4 times higher than that in summer shoots/leaves or newly sprouting turions of the same species [10,12,14]. Mature turions accumulate starch, free sugars, amino acids, reserve proteins, and lipids [15,16,17,18], but the knowledge of the contents of these organic storage substances in turions (except for carbohydrates [14]) is still fragmentary [10]. Starch and free sugars were shown to be the main reserve substances in mature turions of 21 plant species, and their total content ranged from 14 to 63% DW [14]. In contrast, the knowledge on the content and importance of reserve lipids (FAs) in mature turions is still fragmentary. Nevertheless, using light and electron microscopy, Płachno et al. [17] demonstrated the presence of lipid bodies in mature turions in *Aldrovanda vesiculosa* and two *Utricularia* species. The high content of reserve substances makes *Aldrovanda* turions readily eaten by wild ducks [19]. This observation may indicate a significant nutritional value and a lack of toxic components in turions.

To fill the gap in the knowledge, the contents of several FAs known to serve as reserve substances in plants were analyzed in mature turions of 10 species of aquatic plants representing four genera. For the comparison with the turions, the FA contents were also estimated in shoots of summer-growing aquatic plants of four species from two genera. Thus, we tested a hypothesis that aquatic plant turions can be a valuable source of FAs.

## 2. Results

### 2.1. Contents and Composition of Oils

The oil content per dry weight of plant material was about 58% in the shoots (except for *U. vulgaris*, in which it accounted for about 50%) and 43.0, 40.0, and 42.0% in the turions of *A. vesiculosa*, *M. verticillatum*, and *S. aloides*, respectively (Table 1). The oil content in the *Utricularia* turions ranged from 40.7 to 57.0% in *U. australis* and *U. bremii*, respectively. The chromatographic analysis revealed the presence of seven FAs in the analyzed samples (Figures S1–S22 and Table S1), with palmitoleic acid (16:1) identified only in the *U. australis* turions and behenic acid (22:0) only in the *U. bremii* turions. Palmitoleic acid (16:0), stearic acid (18:0), oleic acid (18:1), and linolenic acid (18:2) were found in all the samples. Alpha-linolenic acid (18:3) was present in all the samples except the *S. aloides* oil. Linolenic acid, whose content in the shoots was in the range of 2–5% of DW, was the dominant compound, and a particularly high amount of this compound was found in the *Utricularia* turions (from 19.8 to 34.1% in *U. australis* and *U. stygia*, respectively). Interestingly, the total FA content in the oils isolated from the shoots ranged from about 12 to 28%. A small proportion of FAs was also found in the oils from the *Aldrovanda*, *Myriophyllum*, and *Stratiotes* turions. This indicated the presence of other lipophilic compounds, e.g., membrane lipids (phospho-triglycerides) and steroid lipids. The content of these other lipophilic compounds was very high in the shoots (and some turions), and their proportion to the total oil content was even >85%. In contrast, the FA content of these oils in the *Utricularia* turions (except for *U. intermedia* and *U. vulgaris*) was about 99%, indicating that the turions of these plants contained about 50% of FAs. The source chromatographic data are shown in Figures S1–S22 and Table S1.

**Figure 1:**
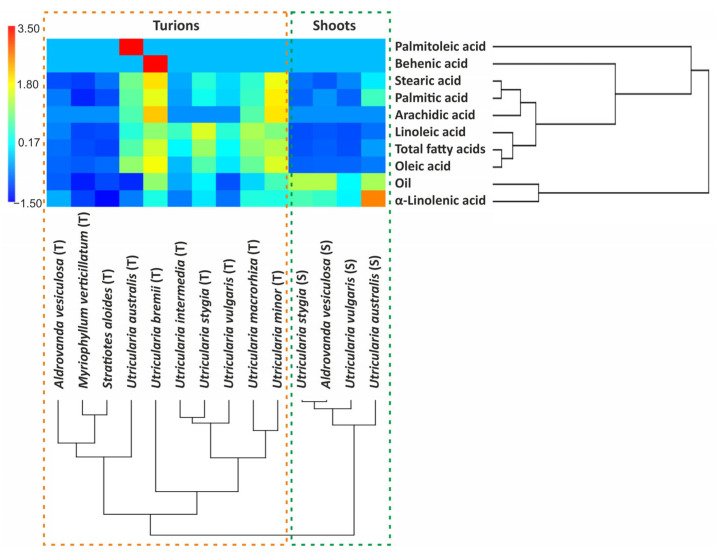
Heatmap and hierarchical cluster analysis dendrogram estimated among 10 turion and 4 shoot samples. Normalized values of the contents of the variables are plotted: red color—high values, blue color—low values.

The dendrogram demonstrated that the lipid composition in the shoots was different from that in the turions, which were grouped into two separate clusters (Figure 1). Three sub-clusters were identified among the turion samples, the first of which contained *A. vesiculosa*, *M. verticillatum*, *S. aloides*, and *U. australis*, the second of which contained *U. bremii* and the third of which contained *U. intermedia*, *U stygia*, *U. vulgaris*, *U. macrorhiza*, and *U. minor*. On the other hand, a lipid similarity analysis identified three main clusters, among which palmitoleic acid was a distinct subcluster, the second subcluster was the total oil and α-linolenic acid, while the third subcluster was the other acids.

### 2.2. Principal Component Analysis

**Figure 2:**
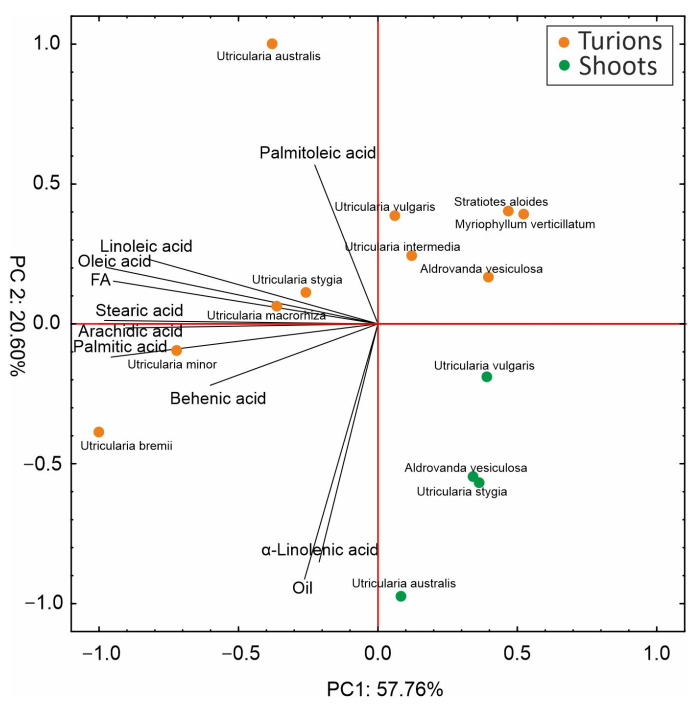
Principal component analysis of the analyzed compounds in the turions and shoots of different species. The length of the lines presents the correlation between the principal component and the original data; FAs—total fatty acids.

The principal component analysis indicated that the first and second components explained almost 58% and 21% of total variations, respectively (Figure 2). Noteworthy is the fact that all variables are negatively correlated with PC1, especially palmitic acid, arachidic acid, stearic acid, oleic acid, linoleic acid, and total FAs, which had a notably negative impact on PC1. On the other hand, it can be noted that α-linolenic acid, oil, and palmitoleic acid more strongly determined PC2, with the first two variables having a negative influence, while palmitoleic acid had a positive impact. The analysis discriminated a few groups of individuals: the first one situated on the left side of the X-axis consists of samples rich in most of the tested metabolites. Here, the turions of five *Utricularia* species, including *U. minor*, *U. macrorhiza*, *U. stygia*, *U. bremii*, and *U. australis*, are found. In turn, on the right side of the X-axis, there are five turion species and four shoot species. However, PC2 facilitates the separation of the turions from the shoots, which are rich in α-linolenic acid and oil in contrast to the aforementioned species.

## 3. Discussion

The large number of FAs in the turions of the analyzed aquatic plants can be explained by the reserve role of these compounds in plants. Since turions are overwintering storage organs from which new plants develop in the spring, they must store energy for the new growth. The energy gathered during photosynthesis can be stored as FAs besides carbohydrates 2. The turions of aquatic plants are exposed to several abiotic stresses, such as anaerobic conditions, transient desiccation, or frost. Fatty acids have been proven to have a great impact on plant adaptation/resistance to several stress factors [20,21,22,23,24]. They also enhance cold tolerance [25,26,27,28,29]. It was shown that several genes, which are involved in the FA synthesis, play critical roles in plant cold tolerance [30,31]. Adamec and Kučerová [32] showed that turions of *Utricularia* and *Aldrovanda* were able to survive mild winter frosts in the topsoil conditions at a rate of 76–100%. These authors proposed that the turions of aquatic species can be hardened by weak frosts to develop frost tolerance. We suggest that this may be connected with the large content of FAs, which are stored in the turion cells of *Utricularia* [17]; however, this suggestion should be proved in future studies. In addition, it has been shown that FAs can form natural deep-eutectic solvents and thus reduce the sensitivity of plants to low temperatures [33]. The differences in the content of FAs between the turions of the studied species can be explained in two ways. They may be related to either the taxonomic position of the examined species (i.e., analyzed species belong to various flowering plant families: *S. aloides* to Hydrocharitaceae, *M. verticillatum* to Haloragaceae, *Utricularia* spp. to Lentibulariaceae, *A. vesiculosa* to Droseraceae) or the different turion physiology and survival strategies of these species. Two different ecological strategies of turion formation, germination, and sprouting can be distinguished [10,11]. The turions of bottom-rooted species form and ripen at depth and germinate and sprout at the bottom. In turn, the turions of submerged rootless and free-floating species form, ripen, germinate, and sprout in the light and warmer water at the surface. These important ecological plant strategies correlated with total non-structural carbohydrate contents in turions of 21 aquatic plant species [14]. In some species, mature turions usually break from the dying mother shoots and sink actively to the bottom, while turions of various *Utricularia* species are usually less dense than water and are dragged temporarily by the dying mother shoots to the bottom. 

It seems that overwintering organs of aquatic carnivorous plants, especially *Utricularia* turions, can be a suitable raw material for obtaining linolenic acid-rich oils. This compound, which belongs to the group of polyunsaturated omega-6 FAs, has important human health-promoting properties. It participates in the biosynthesis of prostaglandins, thromboxanes, and leukotrienes and is a component of lipid membranes. It is believed that linolenic acid supplementation is essential for the proper functioning of the human body [34]. For this reason, *Utricularia* turion oils have significant health-promoting potential. Some limitations may be the availability of turions; therefore, technology should be developed to cultivate these plants and induce their turion production. Overwintering shoots may also be an interesting raw material because of their relatively high content of α-linolenic acid.

There is now a growing interest in various groups of plants (which were previously outside the mainstream of research) as potential sources of chemical organic compounds (including FAs) useful to humans in pharmacy, cosmetology, or food production. For example, terrestrial carnivorous plants from Nepenthales [35,36,37] and Sarraceniaceae [38] have been perceived as a source of important compounds. Thus, our current study using aquatic plants including carnivorous plants (*Utricularia*, *Aldrovanda*) is a part of this trend. The results of our study suggest that aquatic carnivorous plants may become an alternative source of fatty acids to commonly used oilseed crops such as sunflower [39,40], castor bean [41,42], and *Brassica* species [43,44].

## 4. Materials and Methods

### 4.1. Plant Material and Sample Collection

**Figure 3:**
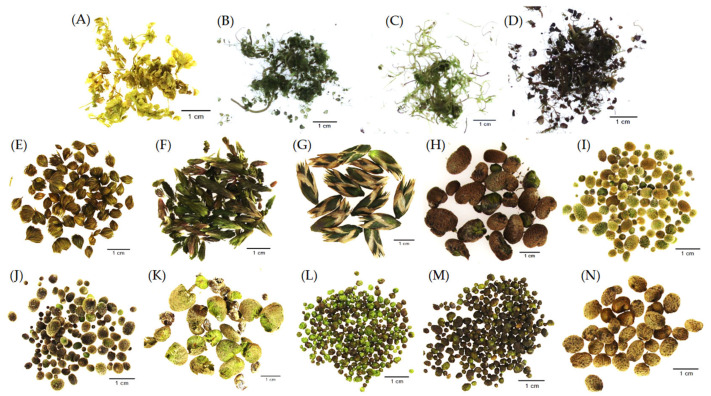
Plant material used in the present study (**A**–**D**)—shoots; (**E**–**N**)—turions. (**A**) Aldrovanda vesiculosa; (**B**) *Utricularia australis;* (**C**) *Utricularia stygia*; (**D**) *Utricularia vulgaris*; (**E**) *A. vesiculosa;* (**F**) *Myriophyllum verticillatum*; (**G**) *Stratiotes aloides*; (**H**) *U. macrorhiza*; (**I**) *U. intermedia*; (**J**) *U. stygia*; (**K**) *U. vulgaris*; (**L**) *U. minor*; (**M**) *U. bremii;* (**N**) *U. australis*.

Fatty acids were analyzed in shoots of summer growing plants of four aquatic species (Figure 3A–D) and in mature turions of 10 aquatic plant species (Figure 3E–N). Approximately 30–60 cm long shoots of *Utricularia australis* were collected from a fishpond in the Třeboň basin, S Bohemia, Czech Republic, and 25–30 cm long apical shoot segments were used for the analyses. Around 16 plants were collected for one sample and three parallel samples were provided. The adult plants of *Utricularia vulgaris* (from Hodonín, Czech Republic, voucher CZ 0 HBT 2017.03644) and *Utricularia stygia* (Třeboň basin, CZ 0 HBT 2017.03767) were collected from a 2.5 m^2^ plastic container with humic water. Fifteen 30–40 cm long apical *U. vulgaris* shoot segments without flowers were collected for each of the two parallel samples. Around 50 plants of *U. stygia* with both photosynthetic and carnivorous shoots (10–15 cm long) were collected for each of the three parallel samples. The adult plants of *A. vesiculosa* (originating from Baláta-tó, SW Hungary, HU 0 HBT 2017.03654) were collected from a 3.5-l outdoor aquaria with humic water (for all types of cultivations, see [45,46,47]. Around 50 4–8 cm long apical shoot segments of *A. vesiculosa* were collected for each of the two parallel samples. All shoots were collected from 5 to 26 July 2021. The mature turions of seven aquatic species (*A. vesiculosa* originating from Lake Długie, E Poland, PL 0 HBT 2017.04079; *Utricularia macrorhiza* from Canada, CA 0 HBT 2017.03544; *U. vulgaris*; *U. stygia*; *Utricularia intermedia*, Třeboň basin, CZ 0 HBT 2017.04058; *Utricularia bremii*, Hluboká nad Vltavou, Czech Rep., CZ 0 HBT 2017.03621; and *Myriophyllum verticillatum*, Mimoň region, Czech Rep., CZ 0 HBT 2017.03638) were collected from outdoor containers of the collection of aquatic plants in the Institute of Botany CAS at Třeboň, while the turions of three species (*U. australis*, *U. minor*, and *Stratiotes aloides*) were collected from natural sites in the Třeboň basin. All plants except for those denoted were collected in the Czech Republic. All turion species created one mixed sample each. All turions were collected from 15 October to 7 November 2021.

The collected plant material was cleaned, washed thoroughly with tap water, blotted dry, transferred into 45 mL pre-weighed glass test tubes, and stored in a freezer at ca. −25 °C for weeks. After lyophilization, the tubes were weighed again and the DW was estimated. The dry weight of each sample exceeded 1 g.

### 4.2. Isolation of Oil and Preparation of Samples for GC Analysis

The plant materials were pulverized and weighed (0.500 g). The samples were extracted four times with hexane (4 × 30 mL) using an ultrasonic bath (4 × 15 min) (Bandelin Sonorex RK 510 H, BANDELIN electronic GmbH & Co., Ltd. KG, Berlin, Germany). The extracts were combined and evaporated in a rotary evaporator (Heidolph HeiVap Expert, Heidolph Instruments GmbH & Co., Ltd. KG, Schwabach, Germany). Weighed oil aliquots (approx. 10 ± 2 mg) were dissolved in 500 µL tert-butyl methyl ether (99.8%) and derivatized by the addition of 250 µL of a 0.25 M methanolic solution of trimethylsulfonium hydroxide. The whole sample was shaken vigorously and the GC analysis was performed. Please find the GC chromatogram in the Supplementary File. 

### 4.3. Fatty Acid Analysis

The oil composition was analyzed according to Strzemski et al. [8]. The analysis was performed using an Agilent GC-MSD system (GC/MSD 6890N/5975) equipped with an HP-88 Agilent capillary column (60 m × 0.25 mm; 0.20 µm film thickness), MSD ChemStation ver. E.02.02.1431 software (Agilent Technologies, Santa Clara, CA, USA), and a split–splitless injector. The oven temperature was programmed from 110 °C to 190 °C with an 8 °C/min gradient held for 2 min at 110 °C and 13 min at 190 °C. The temperature of the injector was 250 °C. The injection volume was 1 µL (split ratio 150:1; split flow 180 mL/min). Helium was used as a carrier gas at a flow rate of 1.2 mL/min. A quadrupole mass spectrometer with electron ionization (EI) at 70 eV and with a full scan-type acquisition mode (50 *m*/*z* to 500 *m*/*z*) was used as a detector connected with the GC. The temperature of the MS source and the MS quadrupole was set to 230 °C and 150 °C, respectively. The identification of the constituents was based on a comparison of their mass spectra with the mass spectra library NIST resources and retention times with standards. Quantitative determinations were made using the internal standard calibration curve method, converting the determined content of each methyl ester to the acid content in 100 g of oil. The percentage content of each acid in the dry plant material was then calculated. Each sample was measured in three analytical replicates [8]. 

### 4.4. Statistical Analysis

The phytochemical relationship between the individuals was visualized as a dendrogram prepared using the unweighted pair group method of arithmetic average (UPGMA) with a Pearson correlation similarity index (PAST4.02 software, version 4.13, Oslo, Norway [48]). The analyses were performed on previously standardized data using the Statistica ver. 13.3 (Tibco Software Inc., Palo Alto, CA, USA). Additionally, the Statistica software was used for principal component analysis.

## 5. Conclusions

Our study shows that the dominant compound in all analyzed plant samples was linolenic acid. The oil content was different in turions and shoots. The oil content in the shoots was higher than that in the turions, but the proportion of fatty acids in the oils from the shoots was low in contrast to those from the turions. The turions of analyzed *Utricularia* consisted of about 50% fatty acids. Thus, our study confirms the suitability of carnivorous plants to be used as a raw material containing organic compounds (especially FAs) useful for humans. However, the novelty of our work is supported by the fact that aquatic *Utricularia* spp. have been omitted as a potential source of important compounds so far. Further research should be conducted to correlate the variation in the FA amounts in turions and low-temperature stress to optimize FA contents. In addition, it is necessary to determine what other lipophilic compounds are components of the oils, since FAs in some oils (especially from shoots) make up only a small percentage.

## Figures and Tables

**Table 1:** Content of oil and fatty acids in aquatic plant shoots (% m/m ± SD; n = 3) and turions (% m/m).

Species	Oil (% of Dry Weight Plant Material)	% of Dry Weight Plant Material								
		Palmitic Acid	Palmitoleic Acid	Stearic Acid	Oleic Acid	Linoleic Acid	Arachidic Acid	α-Linolenic Acid	Behenic Acid	Total Fatty Acids
Shoots										
*Aldrovanda vesiculosa*	58.5 ± 1.00	2.10 ± 0.05	n.d.	0.21 ± 0.18	0.69 ± 0.07	3.24 ± 0.04	n.d.	2.06 ± 0.10	n.d.	8.30 ± 0.09
*Utricularia australis*	58.0 ± 6.87	4.7 ± 0.60	n.d.	0.88 ± 0.06	1.42 ± 0.14	5.24 ± 0.62	n.d.	4.27 ± 0.33	n.d.	16.51 ± 0.35
*Utricularia stygia*	58.5 ± 2.55	1.35 ± 0.22	n.d.	0.31 ± 0.05	0.60 ± 0.17	2.47 ± 0.15	n.d.	2.19 ± 0.59	n.d.	6.92 ± 0.24
*Utricularia vulgaris*	50.3 ± 8.75	1.34 ± 0.01	n.d.	0.41 ± 0.05	0.76 ± 0.09	2.33 ± 0.18	n.d.	1.63 ± 0.35	n.d.	6.47 ± 0.14
Turions										
*Aldrovanda vesiculosa*	43.0	1.72	n.d.	0.15	0.75	6.62	n.d.	1.13	n.d.	10.37
*Myriophyllum verticillatum*	40.0	0.38	n.d.	0.08	0.40	1.78	n.d.	0.39	n.d.	6.03
*Stratiotes aloides*	42.0	0.97	n.d.	0.32	0.89	2.37	n.d.	n.d.	n.d.	4.55
*Utricularia australis*	40.7	5.21	1.60	1.48	10.69	19.75	0.58	0.81	n.d.	40.12
*Utricularia bremii*	57.0	7.27	n.d.	2.26	13.95	28.04	1.29	1.86	1.89	56.56
*Utricularia intermedia*	46.5	2.29	n.d.	0.58	3.61	21.47	n.d.	0.80	n.d.	28.75
*Utricularia macrorhiza*	48.5	4.64	n.d.	1.18	9.17	30.44	0.60	1.90	n.d.	47.93
*Utricularia minor*	53.5	7.90	n.d.	2.05	13.06	26.93	1.20	1.87	n.d.	53.01
*Utricularia stygia*	50.0	3.96	n.d.	1.14	8.55	34.13	n.d.	1.52	n.d.	49.30
*Utricularia vulgaris*	42.5	3.12	n.d.	0.80	5.13	20.03	n.d.	0.73	n.d.	29.81

n.d.—no detection.

## Data Availability

Dataset available on request from the authors.

## Supplementary Materials

The following supporting information can be downloaded at: https://www.mdpi.com/article/10.3390/molecules29092062/s1, Figure S1: GC chromatogram of *Utricularia australis* shoot oil; Figure S2: GC chromatogram of *Aldrovanda vesiculosa* shoot oil; Figure S3: GC chromatogram of *Utricularia stygia* shoot oil; Figure S4: GC chromatogram of *Utricularia vulgaris* shoot oil; Figure S5: GC chromatogram of *Myriophyllum verticillatum* turions oil; Figure S6: GC chromatogram of *Stratiotes aloides* turions oil; Figure S7: GC chromatogram of *Utricularia macrorhiza* turions oil; Figure S8: GC chromatogram of *Utricularia australis* turions oil; Figure S9: GC chromatogram of *Utricularia minor* turions oil; Figure S10: GC chromatogram of *Utricularia bremii* turions oil; Figure S11: GC chromatogram of *Aldrovanda vesiculosa* turions oil; Figure S12: GC chromatogram of *Utricularia intermedia* turions oil; Figure S13: GC chromatogram of *Utricularia stygia* turions oil; Figure S14: GC chromatogram of *Utricularia vulgaris* turions oil; Figure S15: Mass spectrum of palmitic acid methyl ester; Figure S16: Mass spectrum of stearic acid methyl ester; Figure S17: Mass spectrum of oleic acid methyl ester; Figure S18: Mass spectrum of linoleic acid methyl ester; Figure S19: Mass spectrum of alfa-linolenic acid methyl ester; Figure S20: Mass spectrum of arachidic acid methyl ester; Figure S21: Mass spectrum of behenic acid methyl ester; Figure S22: Mass spectrum of palmitoleic acid methyl ester; Table S1: Retention times of fatty acid esters identified in oils and shoots of aquatic plants.
