# Supplementary material for: Shoots and Turions of Aquatic Plants as a Source of Fatty Acids
Source: Molecules. 2024 Apr 29;29(9):2062. doi: 10.3390/molecules29092062 (PMC11085451; doi:10.3390/molecules29092062)
Supplement: Supplementary file 1 [file molecules-29-02062-s001.zip › molecules-2953045-supplementary.pdf]

molecules

Supplementary materials

Shoots and Turions of Aquatic Plants as a Source of Fatty Acids

Maciej Strzemski [1,*], Lubomir Adamec [2], Sławomir Dresler [1,3], Barbara Mazurek [4], Katarzyna Dubaj [5], Piotr Stolarczyk [6], Marcin Feldo [7] and Bartosz J. Płachno [8,*]

1 Department of Analytical Chemistry, Medical University of Lublin, 4a Chodzki St., 20-093 Lublin, Poland; slawomir.dresler@umlub.pl
2 Department of Experimental and Functional Morphology, Institute of Botany of the Czech Academy of Sciences, Dukelská 135, CZ-379 01 Třeboň, Czech Republic; lubomir.adamec@ibot.cas.cz
3 Department of Plant Physiology and Biophysics, Institute of Biological Sciences, Faculty of Biology and Biotechnology, Maria Curie-Skłodowska University, 19 Akademicka St., 20-033 Lublin, Poland
4 Analytical Department, New Chemical Syntheses Institute, 13A Tysiąclecia Państwa Polskiego Ave., 24-110 Puławy, Poland; barbara.mazurek@ins.lukasiewicz.gov.pl
5 Department of Basic Medical Sciences, Faculty of Medical and Health Sciences, Casimir Pulaski Radom University, 27 Boleslawa Chrobrego Str., 26-600 Radom, Poland; k.dubaj@urad.edu.pl
6 Department of Botany, Physiology and Plant Protection, Faculty of Biotechnology and Horticulture, University of Agriculture in Kraków, 29 Listopada 54 Ave., 31-425 Cracow, Poland; piotr.stolarczyk@urk.edu.pl
7 Department of Vascular Surgery, Medical University of Lublin, Staszica 11 St., 20-081 Lublin, Poland; martinf@interia.pl
8 Department of Plant Cytology and Embryology, Institute of Botany, Faculty of Biology, Jagiellonian University in Kraków, 9 Gronostajowa St., 30-387 Krakow, Poland

* Correspondence: maciej.strzemski@umlub.pl (M.S.), bartosz.plachno@uj.edu.pl (B.J.P.)

Table S1. Retention times of fatty acid esters identified in oils and shoots of aquatic plants.

Retention time (min)	Name of fatty acid
24.59	Palmitic acid
25.84	Palmitoleic acid
29.05	Stearic acid
30.05	Oleic acid
31.71	Linoleic acid
33.20	Arachidic acid
33.63	alfa-Linolenic acid
37.16	Behenic acid

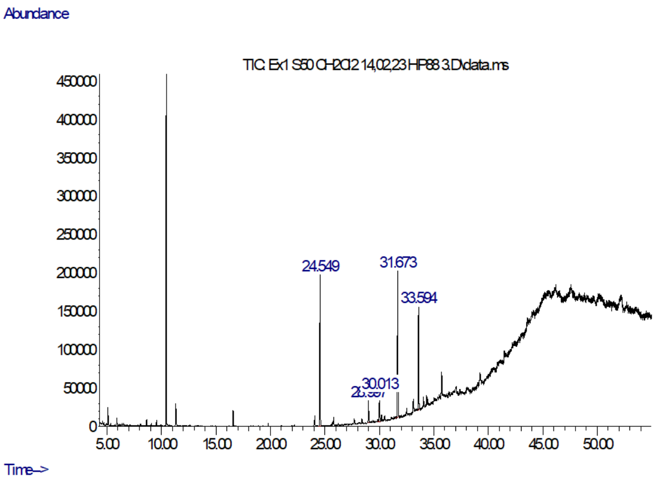

Figure S1. GC chromatogram of *Utricularia australis* shoot oil.

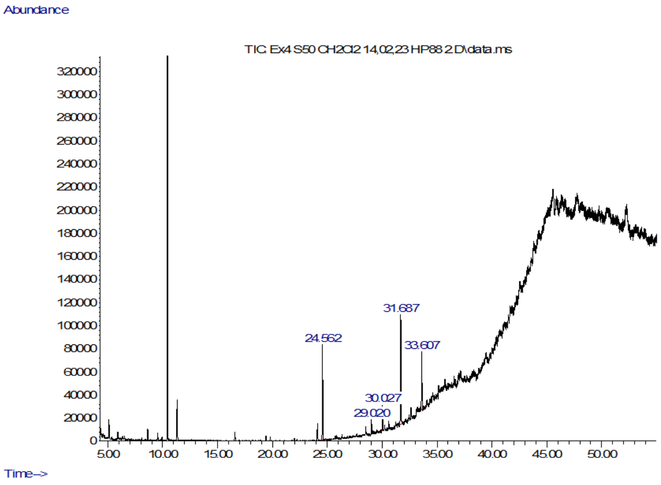

Figure S2. GC chromatogram of *Aldrovanda vesiculosa* shoot oil.

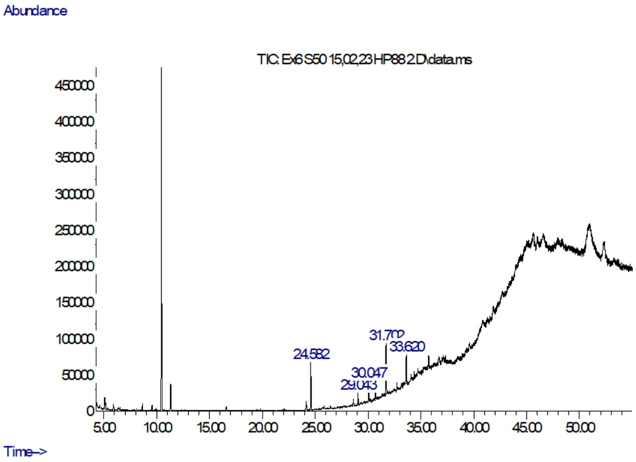

Figure S3. GC chromatogram of *Utricularia stygia* shoot oil.

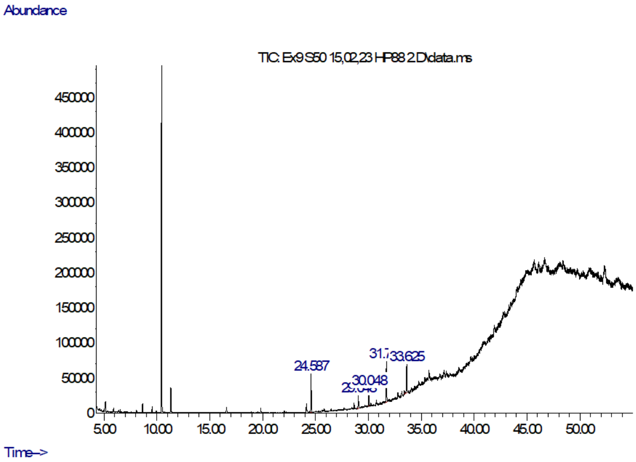

Figure S4. GC chromatogram of *Utricularia vulgaris* shoot oil.

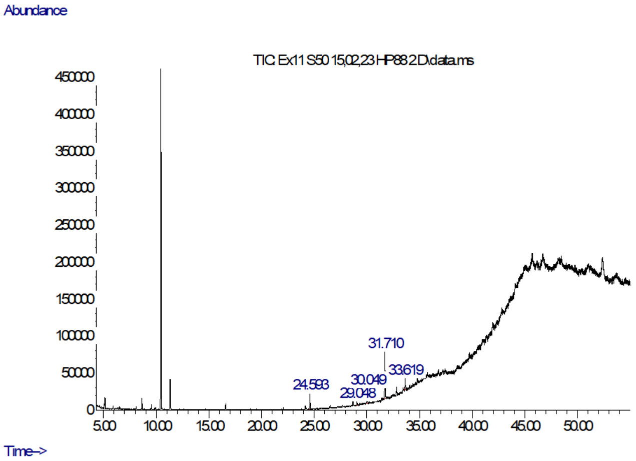

Figure S5. GC chromatogram of *Myriophyllum verticillatum* turions oil.

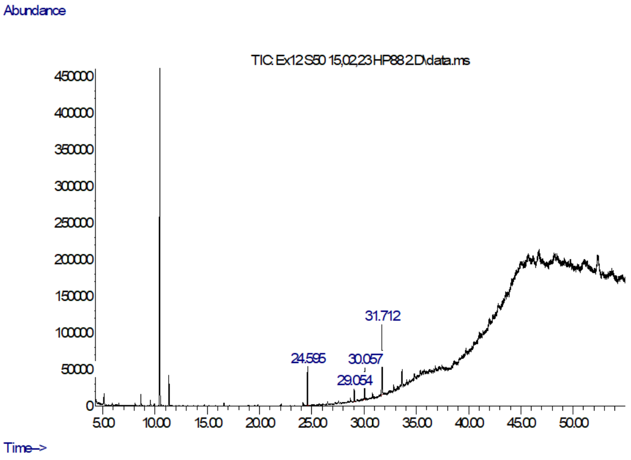

Figure S6. GC chromatogram of *Stratiotes aloides* turions oil.

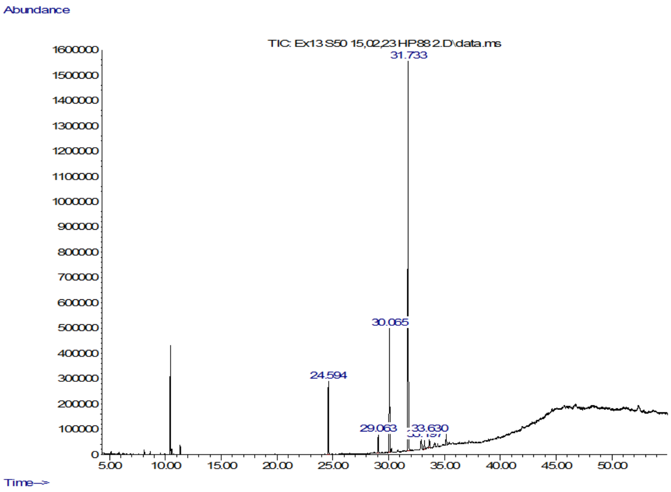

Figure S7. GC chromatogram of *Utricularia macrorhiza* turions oil.

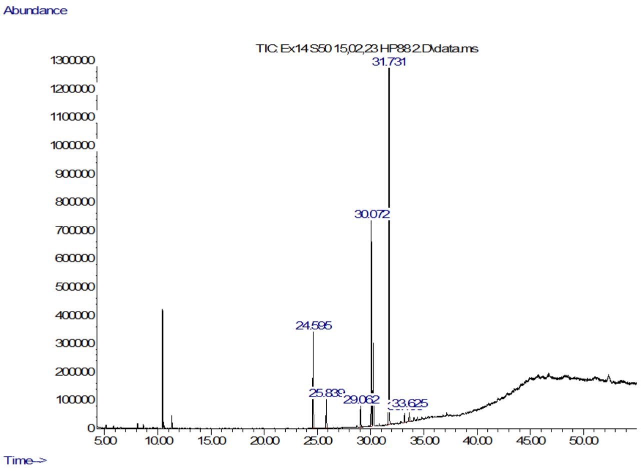

Figure S8. GC chromatogram of *Utricularia australis* turions oil.

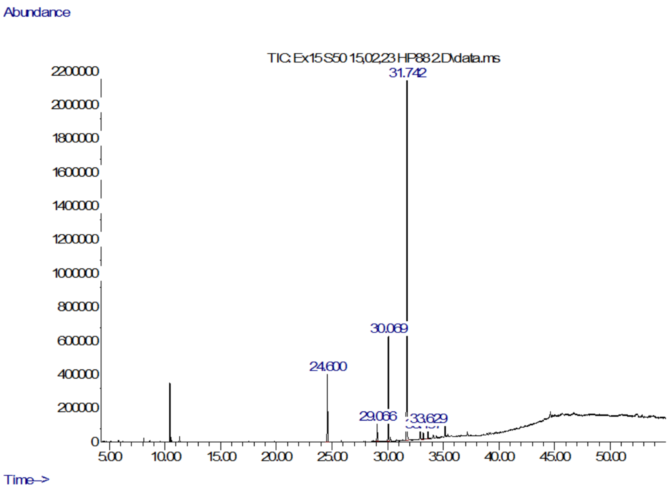

Figure S9. GC chromatogram of *Utricularia minor* turions oil.

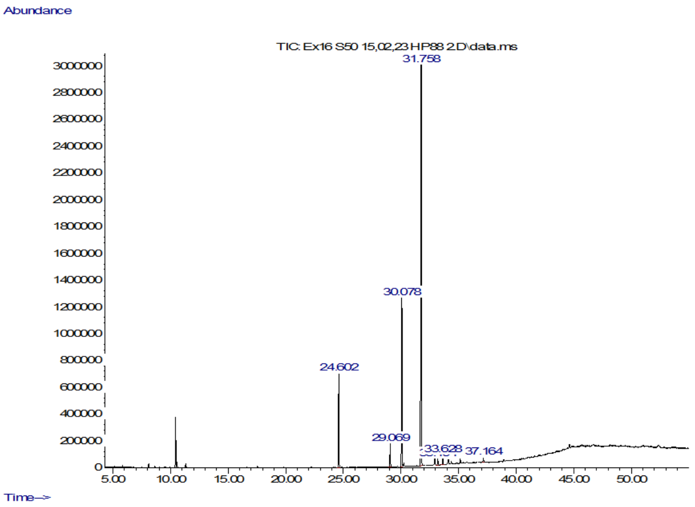

Figure S10. GC chromatogram of *Utricularia bremii* turions oil.

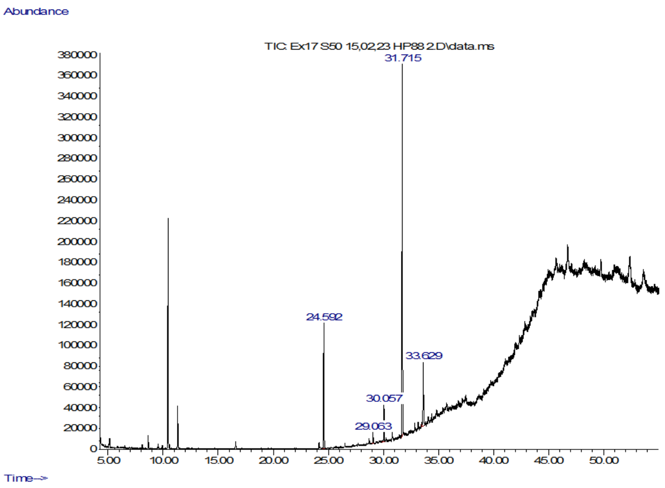

Figure S11. GC chromatogram of *Aldrovanda vesiculosa* turions oil.

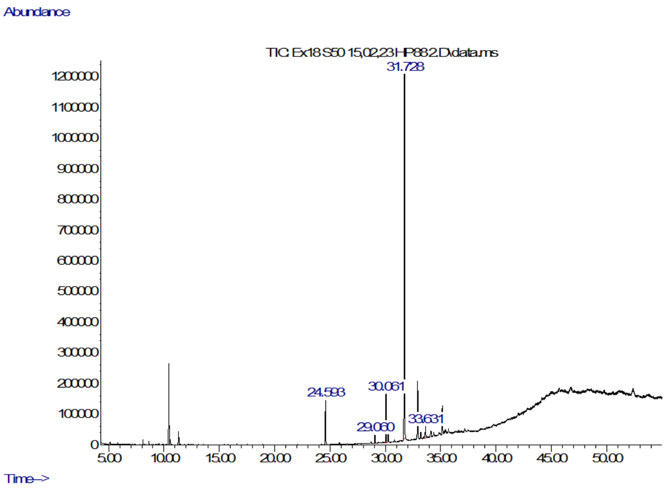

Figure S12. GC chromatogram of *Utricularia intermedia* turions oil.

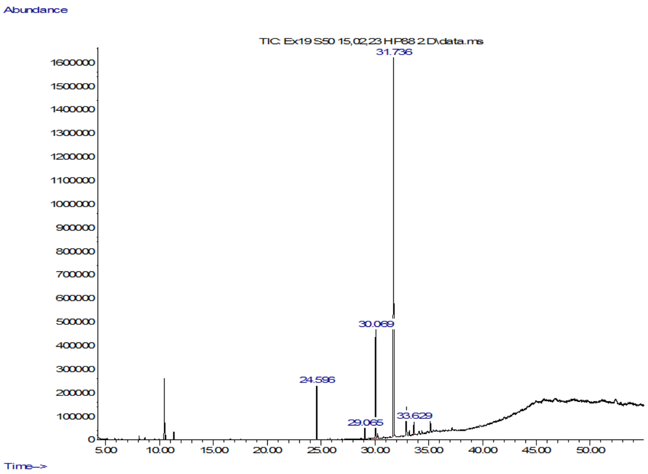

Figure S13. GC chromatogram of *Utricularia stygia* turions oil.

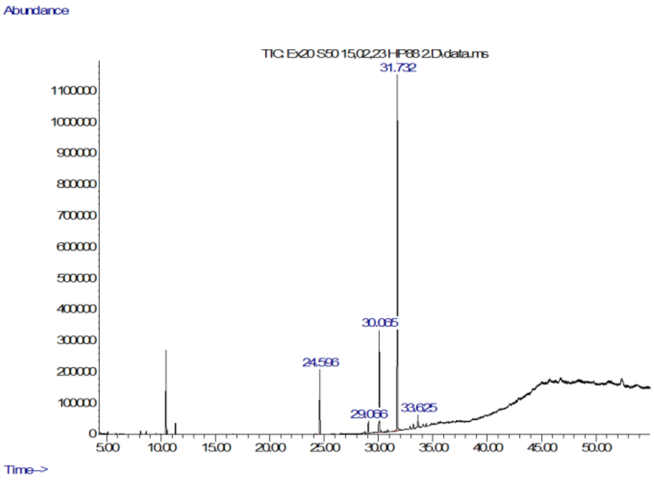

Figure S14. GC chromatogram of *Utricularia vulgaris* turions oil.

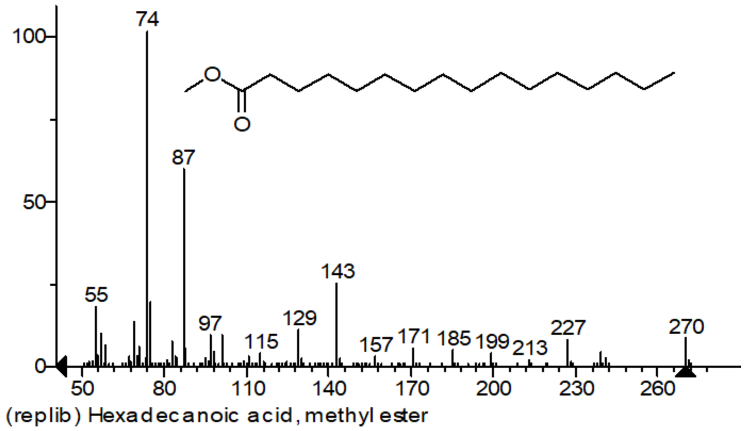

Figure S15. Mass spectrum of palmitic acid methyl ester.

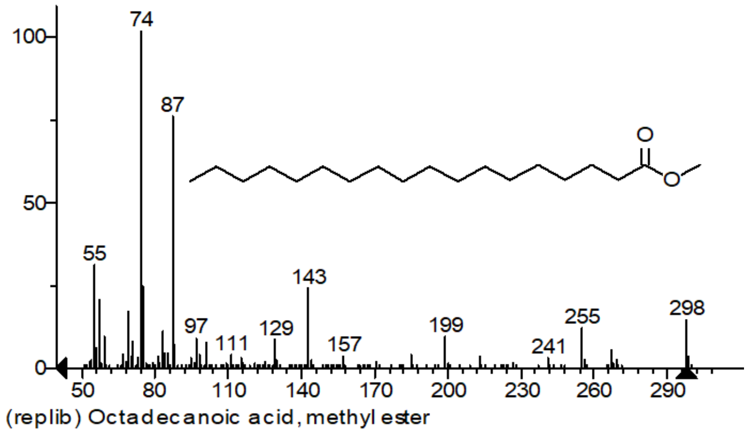

Figure S16. Mass spectrum of stearic acid methyl ester.

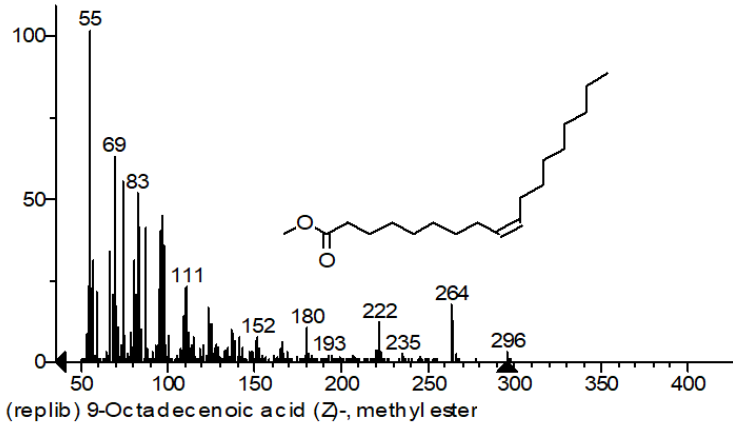

Figure S17. Mass spectrum of oleic acid methyl ester.

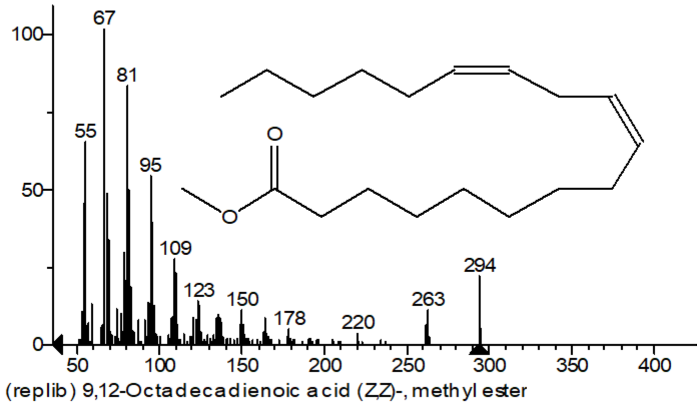

Figure S18. Mass spectrum of linoleic acid methyl ester.

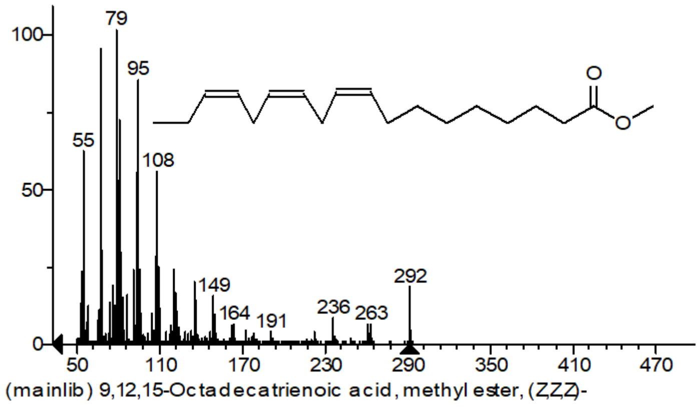

Figure S19. Mass spectrum of alfa-linolenic acid methyl ester.

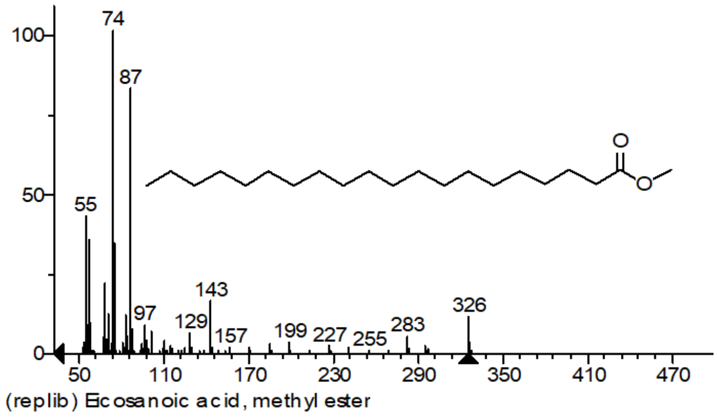

Figure S20. Mass spectrum of arachidic acid methyl ester.

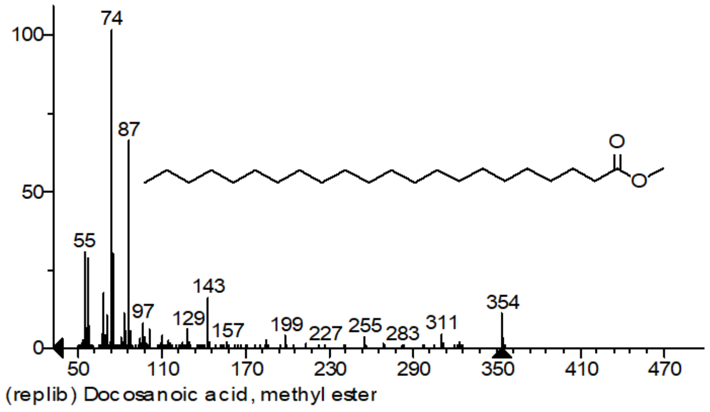

Figure S21. Mass spectrum of behenic acid methyl ester.

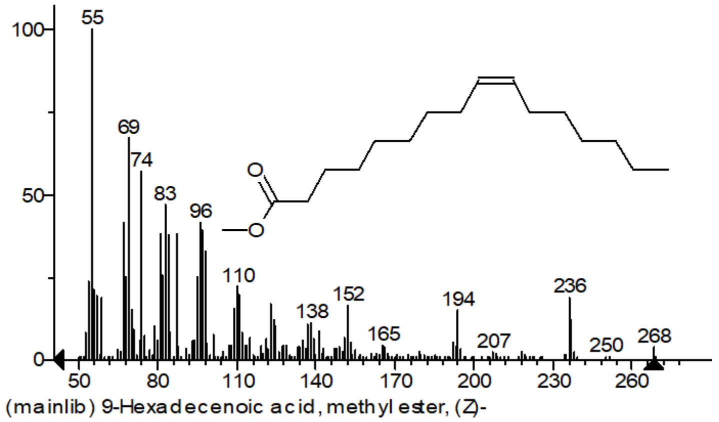

Figure S22. Mass spectrum of palmitoleic acid methyl ester.
